# Patient-Reported Receipt of Oncology Clinician-Delivered Brief Tobacco Treatment (5As) Six Months following Cancer Diagnosis

**DOI:** 10.1159/000528963

**Published:** 2023-03-09

**Authors:** Sarah N. Price, Jordan M. Neil, Melissa Flores, Colin Ponzani, Alona Muzikansky, Lauren Ballini, Jamie S. Ostroff, Elyse R. Park

**Affiliations:** a Department of Psychology, University of Arizona, Tucson, AZ, USA;; b Department of Social Sciences and Health Policy, Wake Forest University School of Medicine, Winston-Salem, NC, USA;; c Departments of Psychiatry and Medicine, Massachusetts General Hospital/Harvard Medical School, Boston, MA, USA;; d Department of Family and Preventive Medicine, University of Oklahoma Health Sciences Center, Oklahoma City, OK, USA;; e Health Promotion Research Center, Stephenson Cancer Center, Oklahoma City, OK, USA;; f Department of Community Health, Tufts University, Medford, MA, USA;; g Department of Psychiatry and Behavioral Sciences, Memorial Sloan Kettering Cancer Center, New York, NY, USA

**Keywords:** Tobacco treatment, Cancer patients, Oncologist, Smoking cessation

## Abstract

**Introduction:**

Smoking after a cancer diagnosis represents a modifiable health risk. It is recommended that oncology clinicians address tobacco use among their patients using the 5As brief model: Asking about use, Advising users to quit, Assessing willingness to quit, Assisting in quit attempts (counseling and medication), and Arranging follow-up. However, cross-sectional studies have found limited adoption of 5As (especially Assist and Arrange) in oncology settings. Further investigation is needed to understand changes in, and factors associated with, 5As delivery over time.

**Methods:**

Patients recently diagnosed with cancer and reporting current smoking (*N* = 303) enrolled in a smoking cessation clinical trial and completed three longitudinal surveys; at pre-intervention baseline and 3- and 6-month follow-up post-enrollment. Patient-level correlates of 5As receipt at baseline, 3 months, and 6 months were identified using multilevel regression models.

**Results:**

At baseline, patient-reported rates of 5As receipt from oncology clinicians ranged from 85.17% (Ask) to 32.24% (Arrange). Delivery declined from baseline to 6-month follow-up for all 5As, with the largest declines observed for Ask, Advise, Assess, and Assist-Counseling. Diagnosis of a smoking-related cancer was associated with greater odds of 5As receipt at baseline but lower odds at 6-month follow-up. At each time point, female gender, religiosity, advanced disease, cancer-related stigma, and smoking abstinence were associated with lower odds of 5As receipt, while reporting a recent quit attempt prior to enrollment was associated with higher odds of 5As receipt.

**Conclusion:**

Oncology clinicians’ 5As delivery declined over time. Clinician delivery of the 5As varied based on patients’ sociodemographics, clinical and smoking characteristics, and psychosocial factors.

## Introduction

Approximately 10–30% of patients with cancer continue to smoke cigarettes post-diagnosis [1, 2]. Continued smoking after a cancer diagnosis is linked to worse quality of life, treatment effectiveness, treatment outcomes, risk of recurrence, risk of developing a second primary cancer, and survival, whereas quitting after diagnosis has the potential to improve these outcomes [3–6].

To address patients’ tobacco use, clinical practice guidelines recommend that healthcare clinicians deliver 5As at every visit (Ask about smoking, Advise cessation, Assess readiness to quit, Assist with motivation and/or cessation, and Arrange follow-up) [7]. Clinical practice guidelines from the National Comprehensive Cancer Network (NCCN) underscore the importance of oncology clinician-delivered smoking cessation interventions, and the American Society of Clinical Oncology (ASCO) includes documentation and counseling of all patients who smoke as a core quality indicator as part of their Quality Oncology Practice Initiative [8, 9]. While there has been some progress as evidenced by increased rates of smoking cessation counseling and referrals from 2015 to 2019, overall rates of treatment and referral remain low (<50%), indicating much room for improvement [10]. To date, the vast majority of studies investigating rates of 5As delivery in oncology settings are cross-sectional and have explored clinician-level (rather than patient level) reporting of 5As delivery [11], limiting understanding of potential inequities in 5A receipt and the degree to which patients who continue to smoke, or have recently quit, receive appropriate follow-up and ongoing cessation support. When queried about barriers to 5As delivery, oncology clinicians commonly cite lack of time and referral pathways [11, 12]. A collaborative care approach (in which patients are referred to a tobacco treatment program embedded in the cancer center and treatment is provided in consultation with oncology care providers) may enhance 5As delivery by minimizing burden on oncology clinicians while still involving them in ongoing smoking cessation treatment [13].

The present study uses data collected from a randomized trial comparing effects of combined pharmacotherapy and intensive smoking cessation counseling to standard tobacco treatment for patients with newly diagnosed cancers in the context of a collaborative care model involving oncologists who provided usual care for patients [14]. Through exploratory secondary analyses of data collected over patients’ 6 months of trial participation, the present study aims to (1) characterize oncology clinicians’ rates and patterns of 5As delivery for recently diagnosed cancer patients during the 6 months following trial enrollment (as reported by patients) and (2) identify patient-level sociodemographic, disease, smoking, and psychosocial factors associated with variation in 5As receipt. The results of this study allow us to explore patient receipt of the 5As in an oncology setting and to identify subpopulations of patients potentially requiring greater attention.

## Patients and Methods

### Participants and Setting

**Fig. 1.**
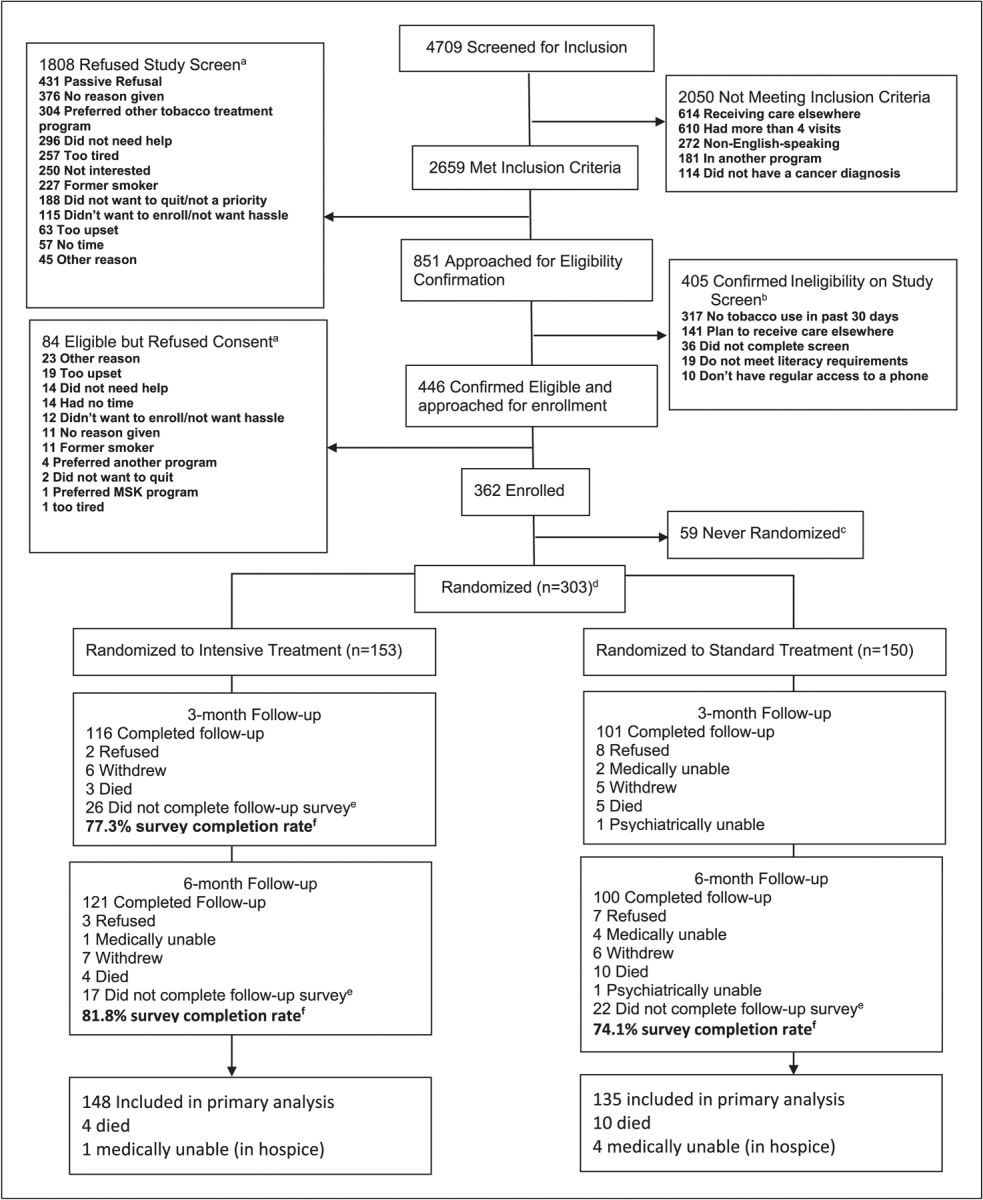
Flowchart of enrollment and intervention to test the effectiveness of two models of tobacco treatment integrated into cancer are at diagnosis. Trial, November 2013 to July 2017. ^a^Patients could give multiple reasons for refusal. The research assistant categorized the reasons of refusal that patients offered according to options available on the study screening tool; those reasons that did not fit into one of these predefined categories were discussed with the team to determine fit with existing categories or establishment of new categories. ^b^Multiple reasons for ineligibility could have been indicated on the screener. As such, the number of reasons exceed the number of patients ineligible. ^c^Those who were never randomized were those who signed a consent form but did not complete a counseling session and were thus not randomized to a treatment group. Reasons included participants who were not able to be reached by the study counselor, participants who withdrew citing other cancer care demands, and participants who became ineligible over time. ^d^A computer was used to randomize participants. Blocks of 6 were used for treatment assignment and were stratified by cancer center clinic and study site. ^e^Those who did not complete follow-up survey include those who were not able to be reached at all to complete the follow-up assessment.^f^Follow-up survey completion rate = completed/completed+ refused+withdrew+did not complete follow-up survey. Participants who were deceased or medically ineligible (e.g., in inpatient hospice or psychiatrically impaired) at follow-up were not included in the final outcome analyses (*n* = 5 intensive treatment and *n* = 15 standard treatment). Thus, for the intensive treatment, the denominator = 148, and for the standard treatment, the denominator = 135.

Data were collected as part of a randomized unblinded clinical trial (NCT01871506). Study design, attrition, power, and cessation outcomes are reported in greater detail elsewhere [14, 15]. Recruitment was standardized across the 2 study sites, Massachusetts General Hospital/Dana Farber Cancer Institute (MGH/DFCI) and Memorial Sloan Kettering Cancer Center (MSKCC). All new patients attending oncology appointments completed a brief screener, and patients were eligible if they self-reported smoking a cigarette, even a puff, within the last 30 days. Beyond self-reported current smoking, eligibility criteria were designed to be as inclusive as possible. Patients had to be at least 18 years old, English- or Spanish-speaking, with a recently diagnosed cancer within 3 months of diagnosis or 4 office visits. Patients were only excluded if they did not plan to receive cancer care at either study site, were psychiatrically or medically unstable, or otherwise unable to provide informed consent as determined by study investigators. Potentially eligible patients were either approached in-clinic or sent an opt-out letter inviting participation (see Fig. 1: CONSORT diagram). This study protocol was reviewed and approved by the MGH/Partners Health System Institutional Review Board (#2013P001036/PHS) and the Memorial Sloan Kettering Cancer Center Institutional Review Board (#14–079) and was conducted in accordance with the Declaration of Helsinki. All subjects provided written informed consent prior to participation.

The study compared intensive tobacco treatment (4 counseling sessions over 1 month, biweekly counseling for 2 months, and 3 monthly booster sessions over 6 months plus choice of free FDA-approved cessation medication) with standard tobacco treatment (4 counseling sessions over 1 month and medication advice). Oncology clinicians were informed of the study and could refer patients but were not required to provide smoking cessation treatment as part of the trial, although delivery of the 5As was encouraged as part of usual care. Both treatments were patient-level interventions using a collaborative care model, whereby oncology clinicians were sent an email notification when a patient started tobacco treatment, encouraging clinicians to provide cessation advice and encouragement. Tobacco treatment specialists delivering the intervention documented all sessions in patients’ electronic health record (EHR), including details regarding tobacco treatment plan, goals, and progress.

### Measures

Participants completed surveys at baseline prior to randomization and at 3- and 6-month post-enrollment follow-up. Baseline surveys were collected in person or by telephone, and follow-up surveys were collected in person or by telephone, mail, or email based on patient preference. Participants self-reported sociodemographics, smoking characteristics, and psychosocial factors relevant to tobacco use and clinical care, while clinical characteristics were abstracted from participants’ medical records.

Sociodemographics assessed at baseline were age, gender, race, ethnicity, education, marital status, and employment. Clinical characteristics assessed at baseline were cancer stage and diagnosis. Based on classification of smoking-related cancers in the 2014 Surgeon General Report [16], we categorized cancer diagnosis into smoking related (e.g., lung, esophageal, head and neck, bladder, kidney, liver, pancreatic, colorectal, anal, small intestinal, gastric, or cervical) and not smoking related (e.g., prostate, testicular, penile, breast, lymphoma, melanoma, or non-cervical gynecological cancer).

Smoking characteristics assessed at baseline included time to first cigarette (a proxy for nicotine dependence), quit attempt history, presence of additional smokers in the household, and smoking-related comorbid disease (including asthma, emphysema/COPD, hypertension, heart attack, stroke). Quit status was determined by patient self-reported continuous abstinence from cigarette smoking at 3 and 6 months, which was biochemically verified (salivary cotinine or expired carbon monoxide) [14].

Psychosocial factors included diagnosis of a serious mental illness (e.g., major depressive disorder, bipolar disorder, schizophrenia), religiosity (assessed at baseline), anxiety and depression symptoms (assessed at all 3 time points), and cancer-related stigma (assessed at baseline and 3 months). Serious mental illness was a binary variable determined by a combination of EHR and patient self-report. Religiosity was measured with a single Likert-style question about the extent to which the respondent considered themselves a religious or spiritual person, ranging from 1 (not religious or spiritual) to 4 (very religious or spiritual). Although use of a single item measure of religiosity/spirituality provides limited understanding, single item measures of religious importance are widely used in contexts where more extensive measurements are not feasible and demonstrate adequate single-item construct validity [17, 18].

Anxiety was measured using the GAD-7 (range 0–21), the sum of 7 items encompassing generalized anxiety disorder symptoms; higher scores indicate greater anxiety [19]. Depression was measured using the PHQ-9 (range 0–27), the sum of 9 items representing symptoms of major depression; higher scores indicate more depressive symptoms [20]. Patient-perceived cancer-related stigma was measured using the sum of 5 items on 4-point Likert scale assessing extent to which patients endorsed feeling internalized shame and blame related to their cancer (range 5–20) [21].

Participant-reported 5As receipt was measured at baseline, 3-and 6-month follow-up in a manner consistent with previous research [22] by asking patients the following question: did your oncology care provider(s) (e.g., doctor, nurse) do any of the following? (1) Ask about your current tobacco use; (2) Advise you to quit; (3) Assess your readiness to quit; (4) Assist you in quitting smoking; and (5) Arrange follow-up. Assist was further separated into (4a) Assist-Talk (talk to you about quitting smoking); (4b) Assist-Counseling (recommend cessation counseling); and (4c) Assist-Medication (recommend nicotine replacement therapy or other cessation pharmacotherapy). Responses to each question were binary (yes/no). The time frame assessed at baseline was “during your last visit,” while the time frame assessed at 3 and 6 months was “in the past 3 months.”

### Statistical Analyses

Analyses were conducted using R version 3.5.1. Table 1 presents descriptive analyses including frequencies, means, and standard deviations for sociodemographic, clinical, and psychosocial variables at baseline. Handling of missing data and attrition are discussed in detail elsewhere [14]. Briefly, scales that were 80% complete were completed with the mean score from available items, and remaining missing data were imputed using a 2-step Markov chain Monte Carlo procedure. At baseline, rates of missing data were relatively low (<5%) for nearly all items, and approximately 30% of the sample was lost to follow-up at 3 and 6 months (Fig. 1). Previous analyses [14] indicated that younger age and more advanced cancer were associated with loss to follow-up at both 3 and 6 months; thus, these factors were included and retained as predictors in all models.

**Fig. 2.**
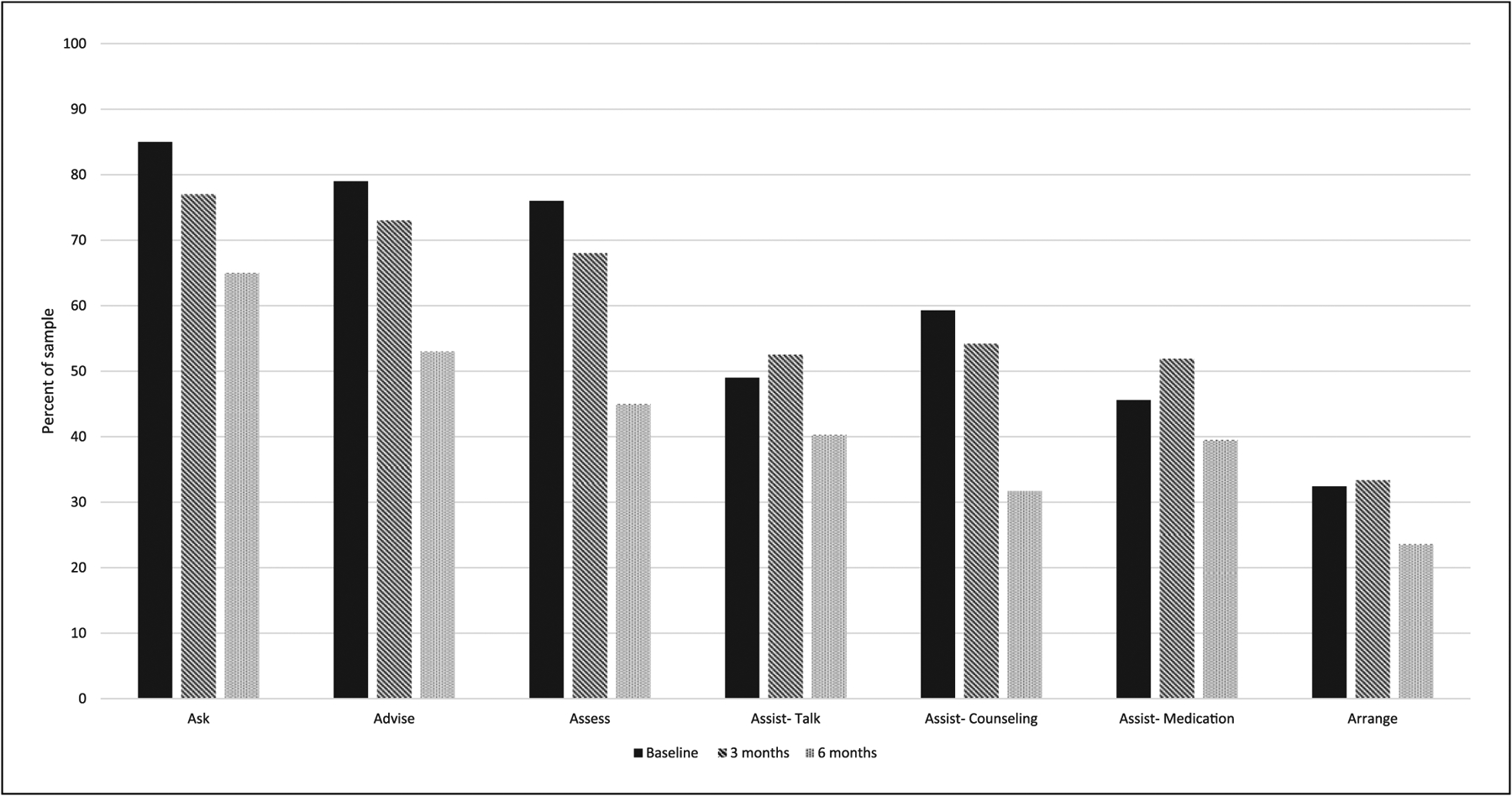
Percentage of sample reporting 5As at each time point. Note: percentages represent proportion of the sample providing data at each time point so as not to under-represent rates of 5As at 3 and 6 months due to loss to follow-up.

Overall rates of oncology clinician 5As delivery at each follow-up were determined by dividing the number of patient participants who reported receiving each of the 5As by the total number of participants providing data for that item for 3-month (217 respondents) and 6-month (221 respondents) follow-up (reported graphically in Fig. 2). While baseline analyses represented pre-intervention 5As as raw percentages [23], we use valid percentages (percent endorsing each A out of the total number providing follow-up data at 3 and 6 months rather than total baseline sample) so as not to under-represent rates of 5As at 3 and 6 months due to loss to follow-up.

To assess the associations between predictors and each of the 5As across the 3 time points, 7 multilevel linear models with time points nested within participants were estimated using a logit link in the *lme4* package [24]. The objective was to create a model for each of the 5As over time, selecting predictor variables for each model in an exploratory manner based on fit and parsimony. Models were built systematically; we first compared the fit of models with a fixed versus random time parameter. Random effects of time did not improve the fit of models; thus, time was modeled only as fixed. Next, we added both between- and within-person fixed effects including study arm, study site, age, cancer stage, and quit status, as well as interactions between each variable and time. We tested interactions with time to determine whether any factors influenced the rate of change in 5As over time. To select variables to include in initial models, Pearson correlations between sociodemographic, clinical, smoking, and psychosocial variables and each of the 5As were examined; those with α levels below 0.10 were included (see Table 2 for a comprehensive list of sociodemographic, clinical, smoking, and psychosocial variables included in initial models and later removed). Level 1 variables (assessed across multiple time points) included in initial models included time, quit status, generalized anxiety, depressive symptoms, and stigma; level 2 variables (assessed only at baseline) included site, arm, age, stage, marital status, time to first cigarette, household smokers, smoking-related cancer, serious mental illness, smoking-related disease, recent quit attempt, and religiosity.

**Fig. 3.**
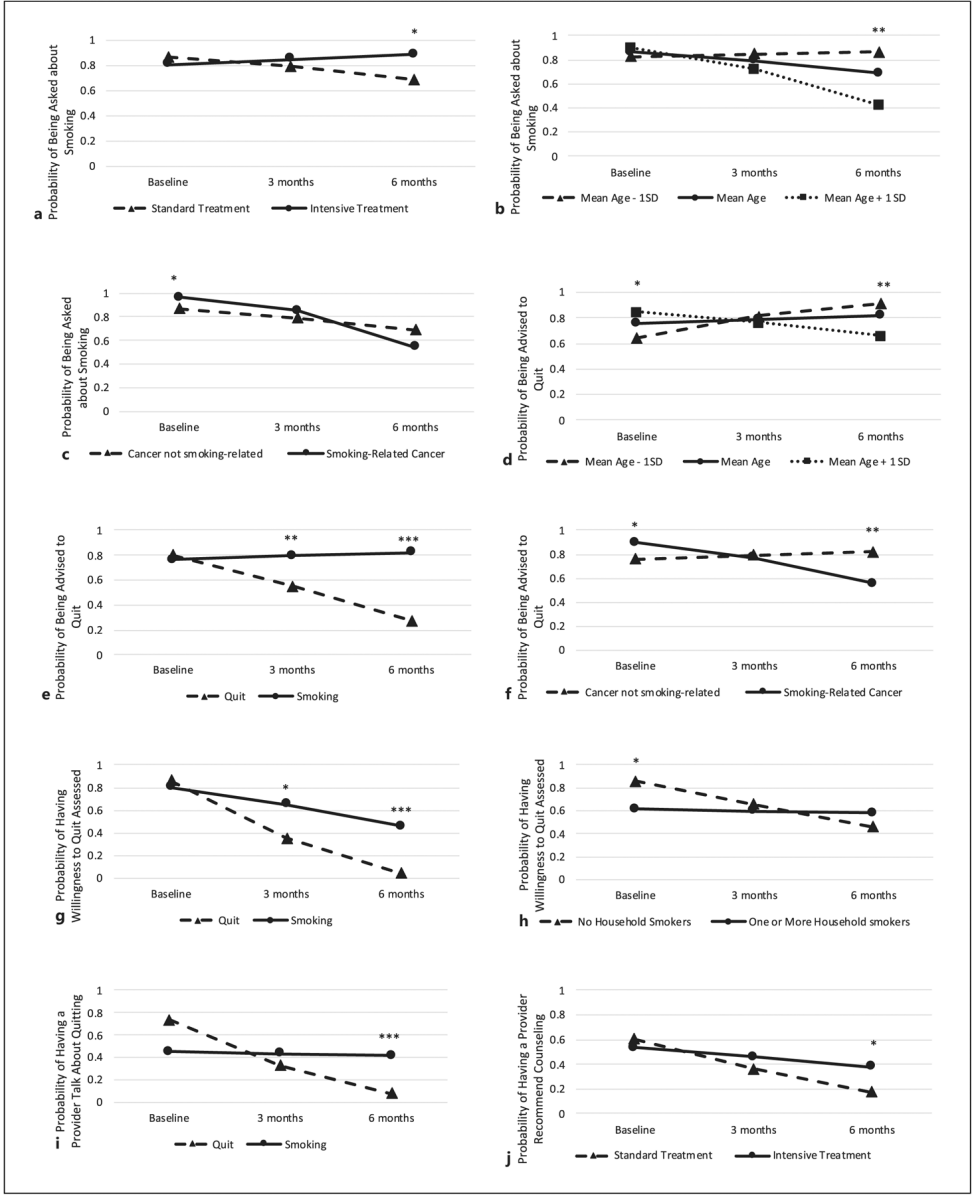
Simple slopes for significant interaction effects. Note: *p ≤ 0.05; **p ≤ 0.01; ***p ≤ 0.001. Each model includes variables listed in Table 3, at the lowest level for categorical variables and at the mean for continuous variables.

Final models for each of the 7 5A variables (presented in Table 3) were then determined based on comparison of fit statistics (i.e., AIC, BIC, model convergence, residuals) of a series of nested models. Through this process of nested model comparison, several variables and interactions were dropped from each model for nonsignificant associations, model fit, and model parsimony. Variables with α ≥ 0.10 were retained in the model if their removal significantly worsened model fit (AIC, BIC), if they were related to study design (e.g., treatment arm, study site), or if they were predictive of attrition (e.g., age, quit status, and cancer stage). All model assumptions and outliers were examined, and models were estimated with and without outliers to test the robustness of effects and consistency of results. Variables included in final models for each of the 5As are included in Table 3. All significant interactions included in the final models were decomposed to obtain simple slopes. As the primary outcomes are binary, simple slopes were transformed from log odds to probabilities for ease of interpretation prior to graphing. Probability graphs for all significant interactions are presented in Figure 3.

## Results

At baseline (N = 303), participants had a mean age of 58 years (SD = 9.71; Table 1). The majority were white (87.46%) and married/partnered (54.13%), with some college education (66.67%). Over half (59.74%) were diagnosed with smoking-related cancers and 48.84% reported a comorbid smoking-related disease. Over one-third (36.60%) were diagnosed with cancer at advanced stages (III or IV). At baseline, participants reported smoking an average of 14 cigarettes per day (SD = 9.89), and 70.60% reported smoking within the first 30 min of waking.

### Rates and Longitudinal Patterns of 5As Delivery

Rates of 5As delivery at baseline (*N* = 303), 3 (*N* = 217), and 6 months (*N* = 221) were as follows: Ask, 85.17%, 77.29%, 65.35%; Advise, 78.62%, 72.5%, 53.47%; Assess, 76.39%, 67.65%, 44.85%; Assist-Talk, 49.29%, 52.5%, 40.31%; Assist-Counseling, 59.29%, 54.19%, 31.79%; Assist-Medication, 45.65%, 51.96%, 39.49%; and Arrange, 32.24%, 33.33%, 23.59%. Overall rates of 5As delivery declined from baseline to 6 months for all 5As, with the largest declines observed for Ask, Advise, Assess, and Assist-Counseling (Fig. 2).

Final models for each of the 5As are presented in Table 3. In all 7 multilevel models estimating 5As across the 3 study time points (baseline, 3 months, and 6 months), time and biochemically verified quit status (quit vs. smoking) were notable and significant predictors of patient-reported receipt of most of the 5As, either as main effects or interactions.

### Predictors of 5As Delivery

#### Ask

In the final model for Ask, there was a main effect of stigma and quit status, such that patients reporting greater cancer-related stigma (OR = 0.90; 95% CI: 0.81, 0.99, *p* = 0.04) and patients who reported being continuously quit between study time points had lower odds of being asked about smoking at each time point (OR = 0.26; 95% CI: 0.12, 0.64, *p* = 0.003; Table 3). There was an interaction between time and treatment arm (*p* = 0.02), age (*p* = 0.002), and smoking-related cancer (*p* = 0.008). Those in the intensive treatment arm were more likely to be asked about smoking at 6 months compared to those in standard treatment (*p* = 0.02), but there was no difference by arm at 3 months (Fig. 3a). Age was not significantly associated with the probability of being asked about smoking at baseline or 3 months, but at 6 months, younger patients were more likely to be asked about smoking (*p* = 0.001; Fig. 3b). Being diagnosed with a smoking-related cancer was associated with a greater probability of being asked at baseline (*p* = 0.01), but not at 3 or 6 months (Fig. 3c).

#### Advise

In the final model for Advise, there was a main effect of cancer stage; patients with more advanced cancer had lower odds of being advised to quit at each time point (OR = 0.43; 95% CI: 0.23, 0.79, *p* = 0.007; Table 3). There was an interaction between time and age (*p* < 0.001), quit status (*p* = 0.03), and smoking-related cancer (*p* < 0.001). While older (vs. younger) patients were more likely to be advised to quit at baseline (*p* = 0.02), by 6 months, older patients were less likely to be advised to quit (*p* = 0.003; Fig. 3d). The probability of being advised to quit remained relatively consistent over time for patients who reported smoking between study time points, but those reporting continuous abstinence were less likely to be advised to quit at 3 (*p* = 0.009) and 6 (*p* < 0.001) months (Fig. 3e). While patients with a smoking-related cancer were more likely to be advised to quit at baseline (*p* = 0.02), by 6 months, they had a lower probability of being advised compared to those without a smoking related cancer (*p* = 0.008; Fig. 3f).

#### Assess

In the final model for Assess, there was a main effect of recent quit attempt history; patients who attempted to quit within 6 months of study enrollment had higher odds of having their willingness to quit assessed at each time point (OR = 2.21; 95% CI: 1.14, 4.30, *p* = 0.04; Table 3). There was an interaction between time and quit status (*p* = 0.046) and time and household smoker (*p* = 0.044). Patients who reported smoking between time points were more likely to have their willingness to quit assessed at 3 (*p* = 0.01) and 6 months (*p* < 0.001) compared to those who did not (Figure 3g). At baseline, patients without additional household smokers were more likely to have their willingness to quit assessed (*p* = 0.001) but not at 3 or 6 months (Figure 3h).

#### Assist-Talk

In the final model for Assist-Talk, there was a main effect of recent quit attempt history; those who attempted to quit in the 6 months prior to study enrollment had higher odds of having a provider talk to them about quitting smoking at each time point (OR = 1.79; 95% CI: 1.10, 2.91, *p* = 0.02; Table 3). There was also a significant quit status by time interaction (*p* = 0.01). Although there was no significant effect of quit status at 3 months, by 6 months, patients who reported smoking between time points were more likely to have a provider assist them than those who had quit (*p* = 0.002; Fig. 3i).

#### Assist-Counseling

In the final model for Assist-Counseling, there was a main effect of age, quit status, religiosity, stigma, and recent quit attempt history. Patients who were older (OR = 1.04; 95% CI: 1.00, 1.07, *p* = 0.04) and patients who attempted to quit during the 6 months prior to enrollment (OR = 1.84; 95% CI: 1.02, 3.32, *p* = 0.04) had higher odds of having smoking cessation counseling recommended to them at each time point. Patients who reported greater cancer-related stigma (OR = 0.89; 95% CI: 0.82, 0.96, *p* = 0.002), greater religiosity (OR = 0.69; 95% CI: 0.48, 0.97, *p* = 0.03), and those who reported continuous abstinence in between time points (OR = 0.23; 95% CI: 0.11, 0.54, *p* < 0.001) were less likely to have smoking cessation counseling recommended to them at each time point (Table 3). There was a significant interaction effect between time and treatment arm (*p* = 0.02). While there was no difference in the probability of being recommended counseling by treatment arm at 3 months, by 6 months, those in intensive treatment were more likely to have counseling recommended than those in standard treatment (*p* = 0.02; Fig. 3j).

#### Assist-Medication

In the final model for Assist-Medication, there was a main effect for quit status; the odds of being recommended smoking cessation medication were significantly lower for patients who reported continuous abstinence at each time point compared to those who reported smoking in between time points (OR = 0.25; 95% CI: 0.14, 0.44, *p* < 0.001; Table 3). There were no significant interactions in the final model for Assist-Medication.

#### Arrange

In the final model for Arrange, there was a main effect of quit status, gender, religiosity, and stigma; male patients (OR = 1.98; 95% CI: 1.06, 3.69, *p* = 0.03) had higher odds of a provider arranging follow-up at each time point. Those with greater self-reported religiosity (OR = 0.58, 95% CI: 0.40, 0.85, *p* < 0.001), greater cancer-related stigma (OR = 0.92; 95% CI: 0.85, 1.00, *p* = 0.045), or continuous abstinence (OR = 0.38; 95% CI: 0.16, 0.89, *p* = 0.03) had lower odds of having follow-up arranged at each time point (Table 3). There were no significant interactions in the final model for Arrange.

## Discussion

The current study provides important insights into rates, patterns, and predictors of patient-reported 5As delivery and highlights variation in 5As receipt among recently diagnosed cancer patients in a clinical trial using a collaborative care model. Rates of patient-reported 5As receipt at baseline ranged from 85.17% (Ask) to 32.24% (Arrange). These findings align with a general pattern observed in prior studies of usual care across medical specialties [12], and among oncology clinicians in particular [25–27], with Ask and Advise completed routinely (~80%) and Assist and Arrange delivered at much lower rates (<50%). A recent cross-sectional study found that oncologists have some of the lowest rates of 5As adherence among various medical specialties, especially for Assess (73.6%) and Assist (18.6%) [12].

Overall, patient-reported receipt of the 5As in this study declined over 6 months, with the largest declines observed for Ask, Advise, Assess, and Assist-Counseling. The decline in 5As delivery over time can be partially explained by patients’ successful quit attempts since patients reporting continuous abstinence between time points were less likely to receive 5As than those who continued to smoke. Although these declines were observed in the context of a collaborative smoking cessation intervention, a previous study found similar declines in usual care, with rates of asking new oncology patients about smoking dropping from 86% to 60.8% at subsequent visits [28]. While it is encouraging that oncologists were attentive to the clinical needs of cancer patients in this study who continue to smoke, clinicians should continue to ask and advise about current smoking after cessation to prevent relapse, which is common among oncology populations [29, 30]. Indeed, among this study population, approximately one-third of patients who had quit at 3 months relapsed by 6 months, highlighting the need for continued assessment, advice, and assistance for all patients [14].

We also found that more intensive follow-up in the intervention arm may have enhanced patient-reported 5As delivery: while there was no difference in 5As receipt by treatment arm at 3 months, by 6-month follow-up, patients in the intensive treatment arm (regardless of quit status) were more likely to receive Ask and Assist-Counseling compared to those in standard treatment. This suggests that although both arms employed a collaborative care approach, continued contact offered through the intensive treatment arm may have helped sustain oncologist involvement in smoking cessation care [11]. Despite the promise of the collaborative care model in targeting all patients who continue to smoke and enhancing follow-up assessment, we identified several disparities in 5As receipt, controlling for quit status.

Sociodemographic characteristics associated with 5As receipt were gender and age. Extending our previously reported baseline findings [23], female gender was associated with lower odds of Arrange at follow-up, which is consistent with prior research demonstrating that although women are more likely to be asked about smoking, they are equally (or less) likely than men to be recommended a specific treatment or to receive follow-up tobacco treatment [31–33]. Gender differences in receipt of Arrange are concerning given evidence that women may experience greater difficulty maintaining long-term smoking abstinence than men [34]. Older patients were more likely to report receiving Advise at baseline and Assist-Counseling at each time point, but younger patients were more likely to receive Ask and Advise at 6 months. Although provider perceptions of potential benefit of cessation may be driving the differential rates of 5As receipt by patient age and disease stage, quitting tobacco confers numerous benefits regardless of disease stage, age, or tumor type [16]. We observed no differences in patient-reported 5As receipt by race or ethnicity at any time point, which is encouraging given previously identified racial/ethnic disparities in receipt of tobacco cessation intervention [35–37]. These results should nevertheless be considered in the context of limited racial/ethnic diversity of this sample (87.46% white, 93.07% non-Hispanic).

Although baseline analyses identified clinical characteristics (diagnosis of a smoking-related cancer or comorbid smoking-related disease) as increasing a patient’s odds of reporting receipt of Ask, Advise, Assist-Medication, or Arrange [23], patients with a smoking-related cancer actually had a lower probability of being advised to quit at follow-up. This finding was unexpected in the context of our baseline analyses and previous cross-sectional research demonstrating that smoking-related cancer diagnoses typically increase the likelihood that patients receive smoking cessation counseling and assistance [38]. Clinician concerns [39, 40] about not triggering shame or self-blame [41] may be behind their reluctance to broach the topic of tobacco use with patients with smoking-related cancers who have been unable to quit. Patients who report cancer-related stigma may be less likely to bring up smoking with their oncology care providers or experience worse patient-provider communication overall [42], and in turn, clinicians may decide not to discuss smoking due to nihilism, pessimistic attitudes [43], or assumptions that patients are unwilling to quit [44].

Patients who engaged in a quit attempt in the 6 months prior to study enrollment were also more likely to receive 5As (Assess, Assist-Talk, and Assist-Counseling) at each study time point, while those who reported greater cancer-related stigma were less likely to receive Ask, Assist-Counseling, and Arrange at each time point. These findings lend further support to the hypothesis that oncology clinicians are more comfortable engaging patients who are already motivated to quit than they are at broaching the subject with patients who may be perceived as more reluctant to discuss smoking or who may be experiencing stigma related to their cancer and/or smoking status. Empathic communication training for oncology providers may help improve communication for patients who may appear more reluctant to discuss smoking [45]. Lastly, greater self-reported religiosity was associated with lower odds of reporting receiving Assist-Counseling and Arrange at each time point. Although understanding the reasons behind this relationship is beyond the scope of this paper, future research may explore the influence of patients’ self-reported religiosity/spirituality on receipt of tobacco cessation intervention.

Results of this study should be considered within the context of its limitations. First, this study involved exploratory secondary analyses in the context of a randomized tobacco trial, so estimates should be interpreted cautiously to identify areas warranting further study in other oncology settings. Both intervention groups were treated using a collaborative care model approach, albeit with a differential treatment dose. This design limits generalizability to other treatment settings and does not allow us to describe naturalistic changes in 5As delivery or quantify the degree to which EHR documentation and care coordination facilitated (or diminished) oncology clinician 5As delivery over time. While it appears that continued follow-up in the intensive treatment arm *improved* 5A delivery at 6 months, it is also possible that oncology providers relying on tobacco counselors to address smoking could explain the generally low rates of Assess, Assist, and Arrange observed at baseline and throughout the study period. Both study sites are also well-resourced comprehensive cancer centers with preexisting tobacco treatment services. Rates of 5As delivery may differ in other settings without a collaborative care model or other embedded tobacco treatment services. Future research should explore the degree to which a collaborative care approach, in which patients are referred to an in-house tobacco treatment program and treatment is provided by tobacco treatment specialists in consultation with oncology clinicians, could enhance 5As delivery by minimizing burden on oncology clinicians while still involving them in smoking cessation treatment. Additionally, all study variables (smoking status, psychosocial factors, and the 5As) were assessed simultaneously, and assessment of 5As at follow-up was asked over a 3-month time frame, which limits understanding of directionality and which could lead to bidirectional causality and biased recall. Nevertheless, despite these limitations, this study has several notable strengths, including the longitudinal design and the heterogeneity of the sample in terms of age, tumor type, and disease stage. Our use of patient (rather than provider) report to assess the 5As is also a strength based on the limitations of clinician self-assessment [46].

## Conclusions

Patient-reported delivery of the 5As in a collaborative oncology setting was associated with patient smoking status, with the greatest declines in 5As receipt reported by patients who were continuously abstinent from smoking. Even in the case of early quit success, however, patients should receive continued tobacco assessment and cessation support due to high risk for relapse.

Differences in 5As receipt were observed even after controlling for smoking status. Certain characteristics of patients that may be associated with provider perceptions of a patient’s reluctance to discuss smoking (e.g., greater perceived cancer-related stigma, less recent history of quit attempts) or with poorer prognoses (e.g., older age, more advanced disease stage) were associated with lower odds of receiving the 5As among those who continued to smoke. Female patients were also less likely to have follow-up arranged at each time point. These results highlight the importance of encouraging oncology clinicians to initiate and sustain conversations about smoking cessation to promote long-term smoking abstinence.

## Figures and Tables

**Table 1.** Patient characteristics at baseline (*N* = 303)

Variable	M (SD)/*n* (%)	Range
Age, years	58.34 (9.71)	21–86
Gender		
Male	133 (43.89)	
Female	170 (56.11)	
Race		
American Indian/Alaska Native	3 (0.99)	
Asian	2 (0.66)	
Black/African American	31 (10.23)	
White	265 (87.46)	
Other	2 (0.66)	
Ethnicity*		
Not Hispanic/Latino	282 (93.07)	
Hispanic/Latino	11 (3.70)	
Marital/relationship status^c^		
Not married/partnered	131 (43.23)	
Married/partnered	164 (54.13)	
Employment status^c^		
Not full-time employed	196 (64.86)	
Full-time employed	100 (33.00)	
Education^c^		
High school or less	93 (30.69)	
Some college or more	202 (66.67)	
Cigarettes per day	14.08 (9.89)	1–70
Time to first cigarette^c^		
Greater than 30 min after waking	83 (27.39)	
Less than 30 min after waking	214 (70.60)	
Recent quit attempt^c^		
Greater than 6 months ago	119 (39.27)	
Less than 6 months ago	81 (26.70)	
Importance of quitting	9.28 (1.60)	0–10
Household smokers^c^		
None	123 (40.59)	
One or more	93 (30.69)	
Perceived cancer-related stigma	10.43 (3.90)	5–20
Comorbid smoking-related disease		
None	155 (51.16)	
Comorbid smoking-related disease	148 (48.84)	
Smoking-related cancer^a^		
Not smoking-related cancer	122 (40.25)	
Smoking-related cancer	181 (59.74)	
Cancer type		
Thoracic	93 (30.69)	
Breast	77 (25.41)	
Genitourinary	51 (16.83)	
Gastrointestinal	29 (9.57)	
Head and neck	31 (10.23)	
Lymphoma	9 (2.97)	
Gynecological	7 (2.31)	
Melanoma	6 (1.98)	
Cancer stage		
0	17 (6.16)	
I	86 (31.16)	
II	67 (24.28)	
III	53 (19.20)	
IV	53 (19.20)	
Nonsolid indolent	3 (0.99)	
Nonsolid advanced	5 (1.65)	
NA/unknown	19 (6.27)	
Stage of diagnosis^c,b^		
Not advanced	182 (60.07)	
Advanced	111 (36.63)	
5As		
Ask	247 (81.5)	
Advise	228 (75.2)	
Assess	220 (72.6)	
Assist (talk)	139 (45.9)	
Assist (counseling)	166 (54.8)	
Assist (medication)	131 (43.2)	
Arrange	93 (30.7)	
Anxiety (GAD-7)	9.5 (5.9)	0–21
Depression (PHQ-9)	8.6 (5.9)	0–27
Religiosity	2.6 (0.9)	1 −4

a Smoking-related cancer comprised anal, bladder, cervical, colorectal, esophageal, gastric, head and neck, kidney, liver, lung, pancreatic, and small intestine cancer types.

b Cancer stages III and IV solid tumor and nonsolid advanced were bifurcated and classified as advanced stage of diagnosis.

c Not all percentages add up to 100 due to missing data.

**Table 2.** Variables tested in initial multilevel logistic regression models for each of the 5As

*1. Ask*
Time, site, arm
Sociodemographic: age, race/ethnicity^a^
Clinical: stage, smoking-related cancer
Smoking: quit status, smoking-related disease^a^, recent quit attempt
Psychosocial: serious mental illness^a^, generalized anxiety^a^, depressive symptoms^a^, stigma, religiosity
*2. Advise*
Time, site, arm
Sociodemographic: age, race/ethnicity^a^, marital status^a^
Clinical: stage, smoking-related cancer
Smoking: quit status, smoking-related disease^a^, recent quit attempt, time to first cigarette^a^
Psychosocial: generalized anxiety^a^, stigma^a^
*3. Assess*
Time, site, arm
Sociodemographic: age, gender^a^
Clinical: stage
Smoking: quit status, smoking-related disease^a^, recent quit attempt, household smoker
Psychosocial: generalized anxiety^a^, depressive symptoms^a^, stigma^a^, religiosity^a^
*4. Assist-Talk*
Time, site, arm
Sociodemographic: age
Clinical: stage
Smoking: quit status, recent quit attempt
Psychosocial: serious mental illness^a^, generalized anxiety^a^, depressive symptoms^a^
*5. Assist-Counseling*
Time, site, arm
Sociodemographic: age, race/ethnicity^a^
Clinical: stage
Smoking: quit status, smoking-related disease^a^, recent quit attempt, household smoker^a^
Psychosocial: generalized anxiety^a^, depressive symptoms^a^, stigma, religiosity
*6. Assist-Medication*
Time, site, arm
Sociodemographic: age, gender^a^
Clinical: stage, smoking-related cancer^a^
Smoking: quit status, smoking-related disease^a^, recent quit attempt^a^, time to first cigarette^a^, household smoker^a^
Psychosocial: generalized anxiety^a^
*7. Arrange*
Time, site, arm
Sociodemographic: age, race/ethnicity^a^, gender
Clinical: stage
Smoking: quit status, smoking-related disease^a^, recent quit attempt
Psychosocial: generalized anxiety^a^, depressive symptoms^a^, stigma^a^, religiosity

a Variable removed from final model on the basis of model fit and parsimony.

**Table 3.** Variables associated with the 5As across study time points in multi-level logistic regression model

	OR [95% CI]	B_log_	*SE*	*z*	p value
*1. Ask*					
Time	0.58 [0.30, 1.10]	−0.55	0.33	−1.66	0.10
Site	1.54 [0.52, 4.52]	0.43	0.55	0.79	0.43
Arm	0.64 [0.23, 1.81]	−0.44	0.53	−0.84	0.40
Age	0.97 [0.91, 1.03]	−0.03	0.03	−1.03	0.30
Stage	0.60 [0.27, 1.34]	−0.51	0.41	−1.24	0.22
Quit status	0.26 [0.12, 0.64]	1.34	0.46	−2.93	0.003**
Smoking-related cancer	4.26 [1.37, 13.23]	1.45	0.58	2.51	0.01*
Stigma	0.90 [0.81, 0.99]	−0.11	0.05	−2.09	0.04*
Recent quit attempt	1.72 [0.77, 3.86]	0.55	0.41	1.32	0.19
Time × arm	2.38 [1.17, 4.86]	0.87	0.36	2.39	0.02*
Time × age	1.07 [1.03, 1.13]	0.07	0.02	3.08	0.002**
Time × smoking-related cancer	0.36 [0.17, 0.77]	−1.03	0.39	−2.64	0.008**
*2. Advise*					
Time	0.31 [0.09, 1.05]	−1.18	0.63	−1.88	0.06
Site	0.99 [0.44, 2.19]	−0.01	0.41	−0.04	0.97
Arm	1.08 [0.61, 1.92]	0.08	0.29	0.26	0.80
Age	0.94 [0.90, 0.99]	−0.06	0.03	−2.27	0.02*
Stage	0.43 [0.23, 0.79]	−0.85	0.31	−2.72	0.007**
Quit status	1.25 [0.20, 7.73]	0.22	0.93	0.24	0.81
Smoking-related cancer	2.82 [1.20, 6.61]	1.04	0.44	2.38	0.02*
Recent quit attempt	1.53 [0.84, 2.79]	0.42	0.31	1.39	0.17
Time × age	1.07 [1.03, 1.12]	0.07	0.02	3.49	<0.001***
Time × quit status	0.26 [0.07, 0.90]	−1.35	0.64	−2.12	0.03*
Time × smoking-related cancer	0.31 [0.16,0.61]	−1.17	0.34	−3.41	<0.001**
*3. Assess*					
Time	0.09 [0.02, 0.44]	−2.43	0.82	−2.97	0.003**
Site	0.99 [0.41, 2.41]	−0.01	0.45	−0.02	0.98
Arm	1.32 [0.70, 2.49]	0.28	0.32	0.85	0.39
Age	1.01 [0.97, 1.04]	0.01	0.02	0.49	0.62
Stage	0.9 [0.48, 1.72]	−0.10	0.33	−0.31	0.76
Quit status	1.48 [0.17, 12.82]	0.39	1.10	0.36	0.72
Household smoker	0.39 [0.16, 0.94]	−0.95	0.45	−2.10	0.04*
Recent quit attempt	2.21 [1.14, 4.30]	0.79	0.34	2.34	0.02*
Time × quit status	0.20 [0.04, 0.97]	−1.63	0.82	−2.00	0.046*
Time × household smoker	2.06 [1.02, 4.16]	0.72	0.36	2.01	0.044*
*4. Assist-Talk*					
Time	0.17 [0.05, 0.61]	−1.75	0.64	−2.73	0.006**
Site	0.60 [0.31, 1.16]	−0.52	0.34	−1.52	0.13
Arm	1.40 [0.87, 2.26]	0.34	0.24	1.38	0.17
Age	1.02 [0.99, 1.05]	0.02	0.01	1.45	0.15
Stage	0.81 [0.49, 1.33]	−0.21	0.25	−0.84	0.40
Quit status	3.37 [0.55, 20.65]	1.21	0.93	1.31	0.19
Recent quit attempt	1.79 [1.10, 2.91]	0.58	0.25	2.33	0.02*
Time × quit status	0.19 [0.05, 0.67]	−1.68	0.65	−2.56	0.01*
5. *Assist-Counseling*					
Time	0.37 [0.23, 0.60]	−0.99	0.24	−4.08	<0.001***
Site	0.82 [0.36, 1.86]	−0.19	0.42	−0.47	0.64
Arm	0.76 [0.36, 1.60]	−0.28	0.38	−0.73	0.47
Age	1.04 [1.00, 1.07]	0.03	0.02	2.06	0.04*
Stage	1.16 [0.64, 2.11]	0.15	0.30	0.49	0.62
Quit status	0.23 [0.11, 0.54]	−1.43	0.41	−3.45	<0.001***
Religiosity	0.69 [0.48, 0.97]	−0.38	0.18	−2.13	0.03*
Stigma	0.89 [0.82, 0.96]	−0.12	0.04	−3.01	0.002**
Recent quit attempt	1.84 [1.02, 3.32]	0.61	0.30	2.03	0.04*
Time × arm	1.98 [1.09. 3.59]	0.68	0.30	2.25	0.02*
*6. Assist-Medication*					
Time	1.06 [0.85, 1.32]	0.05	0.11	0.49	0.62
Site	0.77 [0.51, 1.17]	−0.26	0.21	−1.24	0.21
Arm	0.82 [0.55, 1.21]	−0.20	0.20	−1.02	0.31
Age	1.00 [0.98, 1.02]	0.00	0.01	0.25	0.80
Stage	0.92 [0.61, 1.38]	−0.09	0.21	−0.41	0.68
Quit status	0.25 [0.14, 0.44]	−1.39	0.30	−4.66	<0.001***
*7. Arrange*					
Time	0.87 [0.64, 1.20]	−0.13	0.16	−0.82	0.41
Site	0.73 [0.31, 1.73]	−0.31	0.44	−0.71	0.48
Arm	0.84 [0.47, 1.51]	−0.18	0.30	−0.59	0.56
Age	1.02 [0.99, 1.05]	0.02	0.02	1.11	0.27
Stage	1.22 [0.67, 2.22]	0.20	0.31	0.64	0.52
Quit status	0.38 [0.16, 0.89]	−0.98	0.44	−2.23	0.03*
Gender	1.98 [1.06, 3.69]	0.68	0.32	2.14	0.03*
Religiosity	0.58 [0.40, 0.85]	−0.54	0.19	−2.84	<0.001***
Stigma	0.92 [0.85, 1.00]	−0.08	0.04	−2.00	0.045*
Recent quit attempt	1.76 [0.97, 3.22]	0.57	0.31	1.85	0.06

Reference category for study site = MGH/DFCI; reference category for study arm = standard treatment; reference category for cancer stage = early stage; reference category for quit status = biochemically verified smoker; reference category for smoking-related cancer = no smoking-related cancer; reference category for recent quit attempt= >6 months ago; reference category for household smoker = no household smokers; reference category for gender = female. OR, odds ratio; CI, confidence interval; B_log,_ log odds; SE, standard error.

* *p* ≤ 0.05.

** *p* ≤ 0.01.

*** *p* ≤ 0.001.

## Data Availability

Data were generated by the authors and are not publicly available to protect patient privacy but are available upon reasonable request from the corresponding author.

## References

[1] Jamal A, Homa DM, O’Connor E, Babb SD, Caraballo RS, Singh T, Current cigarette smoking among adults-United States, 2005–2014. MMWR Morb Mortal Wkly Rep. 2015;64(44):1233–40. 26562061 10.15585/mmwr.mm6444a2

[2] Westmaas JL, Alcaraz KI, Berg CJ, Stein KD. Prevalence and correlates of smoking and cessation-related behavior among survivors of ten cancers: findings from a nationwide survey nine years after diagnosis. Cancer Epidemiol Biomarkers Prev. 2014;23(9):1783–92. 25100826 10.1158/1055-9965.EPI-14-0046

[3] National Center for Chronic Disease Prevention and Health Promotion Office on Smoking and Health. Smoking cessation: a report of the Surgeon general. 2020.

[4] Warren GW, Alberg AJ, Cummings KM, Dresler C. Smoking cessation after a cancer diagnosis is associated with improved survival. J Thorac Oncol. 2020; 15(5):705–8. 32197939 10.1016/j.jtho.2020.02.002

[5] Parsons A, Daley A, Begh R, Aveyard P. Influence of smoking cessation after diagnosis of early stage lung cancer on prognosis: systematic review of observational studies with meta-analysis. BMJ. 2010;340:b5569. 20093278 10.1136/bmj.b5569 PMC2809841

[6] Caini S, Del Riccio M, Vettori V, Scotti V, Martinoli C, Raimondi S, Quitting smoking at or around diagnosis improves the overall survival of lung cancer patients: a systematic review and meta-analysis. J Thorac Oncol. 2022;17(5):623–36. 34995798 10.1016/j.jtho.2021.12.005

[7] Clinical Practice Guideline Treating Tobacco Use and Dependence 2008 Update Panel, Liaisons, and Staff; Jaén CR, Baker TB, Bailey WC, Benowitz NL, Curry SJ. A clinical practice guideline for treating tobacco use and dependence: 2008 update. A U.S. Public health service report. Am J Prev Med. 2008;35(2):158–76. 18617085 10.1016/j.amepre.2008.04.009 PMC4465757

[8] Hanna N, Mulshine J, Wollins DS, Tyne C, Dresler C. Tobacco cessation and control a decade later: American society of clinical oncology policy statement update. J Clin Oncol. 2013;31(25):3147–57. 23897958 10.1200/JCO.2013.48.8932

[9] Shields PG, Bierut LJ, Arenberg D, Balis D, Benowitz NL, Burdalski CE, NCCN guidelines version 1.2021 smoking cessation 2021.

[10] Shapiro CL, Zubizarreta N, Moshier E, Brockway JP, Mandeli J, Markham MJ, Quality care in survivorship: lessons learned from the ASCO quality oncology practice initiative. JCO Oncol Pract. 2021;17(8):e1170–80. 34283637 10.1200/OP.21.00290 PMC8360453

[11] Price SN, Studts JL, Hamann HA. Tobacco use assessment and treatment in cancer patients: a scoping review of oncology care clinician adherence to clinical practice guidelines in the U.S. Oncologist. 2019;24(2): 229–38. 30446582 10.1634/theoncologist.2018-0246 PMC6369951

[12] Schaer DA, Singh B, Steinberg MB, Delnevo CD. Tobacco treatment guideline use and predictors among U.S. Physicians by specialty. Am J Prev Med. 2021;61(6):882–9. 34364726 10.1016/j.amepre.2021.05.014 PMC8608714

[13] Bidassie B, Hoffman-Hōgg L, Eapen S, Aggarwal A, Park YA, Keller A. Cancer care collaborative approach to optimize clinical care. Fed Pract. 2017;34(Suppl 3):S42–9. 31089321 PMC6375583

[14] Park ER, Perez GK, Regan S, Muzikansky A, Levy DE, Temel JS, Effect of sustained smoking cessation counseling and provision of medication vs shorter-term counseling and medication advice on smoking abstinence in patients recently diagnosed with cancer: a randomized clinical trial. JAMA. 2020; 324(14):1406–18. 33048154 10.1001/jama.2020.14581 PMC8094414

[15] Park ER, Ostroff JS, Perez GK, Hyland KA, Rigotti NA, Borderud S, Integrating tobacco treatment into cancer care: study protocol for a randomized controlled comparative effectiveness trial. Contemp Clin Trials. 2016;50:54–65. 27444428 10.1016/j.cct.2016.07.016 PMC5035625

[16] US Department of Health and Human Services. The health consequences of smoking—50 Years of progress: a report of the surgeon general. Atlanta (GA); 2014.

[17] Svob C, Wong LYX, Gameroff MJ, Wickramaratne PJ, Weissman MM, Kayser J. Understanding self-reported importance of religion/spirituality in a North American sample of individuals at risk for familial depression: a principal component analysis. PLoS One. 2019;14(10):e0224141. 31626682 10.1371/journal.pone.0224141 PMC6799910

[18] The Association of Religion Data Archives. Single item measures: religion, importance of n.d. (accessed December 17, 2021).

[19] Spitzer RL, Kroenke K, Williams JB, Löwe B. A brief measure for assessing generalized anxiety disorder: the GAD-7. Arch Intern Med. 2006;166(10):1092–7. 16717171 10.1001/archinte.166.10.1092

[20] Kroenke K, Spitzer RL. The PHQ-9: a new depression diagnostic and severity measure. Psychiatr Ann. 2002;32(9):509–15.

[21] Fife BL, Wright ER. The dimensionality of stigma: a comparison of its impact on the self of persons with HIV/AIDS and cancer. J Health Social Behav. 2000;41(1):50–67. 10750322

[22] Park ER, Gareen IF, Japuntich S, Lennes I, Hyland K, DeMello S, Primary care provider-delivered smoking cessation interventions and smoking cessation among participants in the national lung screening trial. JAMA Intern Med. 2015;175(9): 1509–16. 26076313 10.1001/jamainternmed.2015.2391 PMC5089370

[23] Neil JM, Price SN, Friedman ER, Ponzani C, Ostroff JS, Muzikansky A, Patient-level factors associated with oncology provider-delivered brief tobacco treatment among recently diagnosed cancer patients. Tob Use Insights. 2020;13:1179173X20949270. 10.1177/1179173X20949270 PMC7436840 32874095

[24] Bates D, Mächler M, Bolker BM, Walker SC. Fitting linear mixed-effects models using lme4. J Stat Software. 2015;67:1–48.

[25] Warren GW, Marshall JR, Cummings KM, Toll BA, Gritz ER, Hutson A, Addressing tobacco use in patients with cancer: a survey of American Society of clinical oncology members. J Oncol Pract. 2013;9(5):258–62. 23943904 10.1200/JOP.2013.001025 PMC3770508

[26] Warren GW, Marshall JR, Cummings KM, Toll B, Gritz ER, Hutson A, Practice patterns and perceptions of thoracic oncology providers on tobacco use and cessation in cancer patients. J Thorac Oncol. 2013;8(5): 543–8. 23529191 10.1097/JTO.0b013e318288dc96 PMC3628367

[27] Goldstein AO, Ripley-Moffitt CE, Pathman DE, Patsakham KM. Tobacco use treatment at the U.S. National Cancer Institute’s designated cancer centers. Nicotine Tob Res. 2013;15(1):52–8. 22499079 10.1093/ntr/nts083 PMC3842130

[28] Weaver KE, Danhauer SC, Tooze JA, Blackstock AW, Spangler J, Thomas L, Smoking cessation counseling beliefs and behaviors of outpatient oncology providers. Oncologist. 2012;17(3):455–62. 22334454 10.1634/theoncologist.2011-0350 PMC3316932

[29] Simmons VN, Litvin EB, Jacobsen PB, Patel RD, McCaffrey JC, Oliver JA, Predictors of smoking relapse in patients with thoracic cancer or head and neck cancer. Cancer. 2013;119:1420–7. 23280005 10.1002/cncr.27880 PMC3604135

[30] Berg CJ, Thomas AN, Mertens AC, Schauer GL, Pinsker EA, Ahluwalia JS, Correlates of continued smoking versus cessation among survivors of smoking-related cancers. Psychooncology. 2013;22(4):799–806. 22488864 10.1002/pon.3077 PMC3425712

[31] Farmer MM, Rose DE, Riopelle D, Lanto AB, Yano EM. Gender differences in smoking and smoking cessation treatment: an examination of the organizational features related to care. Womens Health Issues. 2011;21(4 Suppl): S182–9. 21724139 10.1016/j.whi.2011.04.018

[32] Chase EC, Mcmenamin SB, Halpin HA. Medicaid provider delivery of the 5A’s for smoking cessation counseling n. Nicotine Tob Res. 2007 Nov;9(11):1095–101. 17978983 10.1080/14622200701666344

[33] Sherman SE, Fu SS, Joseph AM, Lanto AB, Yano EM. Gender differences in smoking cessation services received among veterans. Womens Health Issues. 2005;15(3):126–33. 15894198 10.1016/j.whi.2005.01.001

[34] Smith PH, Bessette AJ, Weinberger AH, Sheffer CE, McKee SA. Sex/gender differences in smoking cessation: a review. Prev Med. 2016;92:135–40. 27471021 10.1016/j.ypmed.2016.07.013 PMC5085924

[35] Landrine H, Corral I, Campbell KM. Racial disparities in healthcare provider advice to quit smoking. Prev Med Rep. 2018;10:172–5. 29868363 10.1016/j.pmedr.2018.03.003 PMC5984231

[36] Lopez-Quintero C, Crum RM, Neumark YD. Racial/ethnic disparities in report of physician-provided smoking cessation advice: analysis of the 2000 National health interview survey. Am J Public Health. 2006; 96(12):2235–9. 16809587 10.2105/AJPH.2005.071035 PMC1698147

[37] Cokkinides VE, Halpern MT, Barbeau EM, Ward E, Thun MJ. Racial and ethnic disparities in smoking-cessation interventions: analysis of the 2005 National health interview survey. Am J Prev Med. 2008;34(5):404–12. 18407007 10.1016/j.amepre.2008.02.003

[38] Rigotti NA. Smoking cessation in patients with respiratory disease: existing treatments and future directions. Lancet Respir Med. 2013;1(3):241–50. 24429130 10.1016/S2213-2600(13)70063-8

[39] Hamann HA, Ostroff JS, Marks EG, Gerber DE, Schiller JH, Lee SJ. Stigma among patients with lung cancer: a patient-reported measurement model. Psychooncology. 2014; 23(1):81–92. 24123664 10.1002/pon.3371 PMC3936675

[40] Champassak SL, Goggin K, Finocchario-Kessler S, Farris M, Ehtesham M, Schoor R, A qualitative assessment of provider perspectives on smoking cessation counselling. J Eval Clin Pract. 2014;20(3):281–7. 24628799 10.1111/jep.12124 PMC4441728

[41] Stiefel F, Bourquin C. Adverse effects of “teachable moment” interventions in lung cancer: why prudence matters. J Thorac Oncol. 2018;13(2):151–3. 29241673 10.1016/j.jtho.2017.10.018

[42] Shen MJ, Hamann HA, Thomas AJ, Ostroff JS. Association between patient-provider communication and lung cancer stigma. Support Care Cancer. 2016;24(5):2093–9. 26553030 10.1007/s00520-015-3014-0 PMC4805469

[43] Hamann HA, Lee JW, Schiller JH, Horn L, Wagner LI, Chang VT, Clinician perceptions of care difficulty, quality of life, and symptom reports for lung cancer patients: an analysis from the symptom outcomes and practice patterns (SOAPP) study. J Thorac Oncol. 2013;8(12):1474–83. 24189514 10.1097/01.JTO.0000437501.83763.5d PMC3936653

[44] Hamann HA, Ver Hoeve ES, Carter-Harris L, Studts JL, Ostroff JS. Multilevel opportunities to address lung cancer stigma across the cancer control continuum. J Thorac Oncol. 2018;13(8):1062–75. 29800746 10.1016/j.jtho.2018.05.014 PMC6417494

[45] Banerjee SC, Haque N, Bylund CL, Shen MJ, Rigney M, Hamann HA, Responding empathically to patients: a communication skills training module to reduce lung cancer stigma. Transl Behav Med. 2021;11(2):613–8. 32080736 10.1093/tbm/ibaa011 PMC7963287

[46] Davis DA, Mazmanian PE, Fordis M, Van Harrison R, Thorpe KE, Perrier L. Accuracy of physician self-assessment compared with observed measures of competence: a systematic review. JAMA. 2006;296(9):1094–102. 16954489 10.1001/jama.296.9.1094

